# Socioeconomic Position, Multimorbidity and Mortality in a Population Cohort: The HUNT Study

**DOI:** 10.3390/jcm9092759

**Published:** 2020-08-26

**Authors:** Kristin Hestmann Vinjerui, Johan H. Bjorngaard, Steinar Krokstad, Kirsty A. Douglas, Erik R. Sund

**Affiliations:** 1 HUNT Research Centre, Department of Public Health and Nursing, Faculty of Medicine and Health Sciences, NTNU–Norwegian University of Science and Technology, 7600 Levanger, Norway; steinar.krokstad@ntnu.no (S.K.); erik.r.sund@ntnu.no (E.R.S.); 2 Academic Unit of General Practice, Australian National University Medical School, the Australian National University, Canberra 2600, Australian Capital Territory, Australia; kirsty.a.douglas@anu.edu.au; 3 Psychiatric Department, Levanger Hospital, Nord-Trøndelag Hospital Trust, 7601 Levanger, Norway; 4 Faculty of Nursing and Health Sciences, Nord University–Levanger Campus, 7601 Levanger, Norway; johan.h.bjorngaard@ntnu.no; 5 Department of Public Health and Nursing, Faculty of Medicine and Health Sciences, NTNU–Norwegian University of Science and Technology, 7491 Trondheim, Norway; 6 Levanger Hospital, Nord-Trøndelag Hospital Trust, 7601 Levanger, Norway

**Keywords:** multimorbidity, frailty, socioeconomic status, mortality, occupations, public health, health inequality, The HUNT Study

## Abstract

Multimorbidity and socioeconomic position are independently associated with mortality. We investigated the association of occupational position and several multimorbidity measures with all-cause mortality. A cohort of people aged 35 to 75 years who participated in the Trøndelag Health Study in 2006–2008 and had occupational data was linked to the Norwegian National Population Registry for all-cause mortality from study entry until 1 February 2019. Logistic regression models for each occupational group were used to analyze associations between the number of conditions and 10-year risk of death. Cox regression models were used to examine associations between combinations of multimorbidity, occupational position, and mortality. Analyses were conducted for men and women. Included were 31,132 adults (16,950 women (54.4%)); occupational groups: high, 7501 (24.1%); low, 15,261 (49.0%)). Increased mortality was associated with lower occupational group, more chronic conditions, and all multimorbidity measures. The joint impact of occupational group and multimorbidity on mortality was greater in men than women. All multimorbidity measures are strongly associated with mortality, with varying occupational gradients. Social differences in multimorbidity are a public health challenge and necessitate consideration in health care. Men in lower occupational groups seem to be a particularly vulnerable group.

## 1. Introduction

The burdens of disease and death are greater for people in lower socioeconomic positions worldwide [1]. Multimorbidity, the concurrence of multiple chronic conditions [2], is highly prevalent [3,4], while health inequalities are most often studied in association with individual diseases.

Multimorbidity may co-occur with frailty [5,6], which is a dynamic, multidimensional symptom complex of accumulated decline in homeostatic reserves that results in increased vulnerability [4]. Both concepts are proxy measures of biological aging [7].

Numerous operational definitions and differences in methods and settings hamper the comparability of research on multimorbidity [8,9,10] and frailty [6,11]. Still, in cross-sectional studies, acknowledged common determinants associated with multimorbidity and frailty are socioeconomic deprivation [8,12], female sex [8,13], and higher age [8,13]. In Norway, previous studies on complex measures of multimorbidity, including frailty, reproduced increased prevalence in people in lower occupational groups, women, and older adults [14,15]. While the prevalence of multimorbidity and frailty rise with higher age, those younger than 65 years encompass a larger number of individuals with multimorbidity and frailty [15,16,17,18].

A review of 26 cohort studies in populations older than 65 years established that, despite heterogeneity, any multimorbidity increases mortality [19]. Only five studies adjusted for sociodemographic variables which reduced the effect estimates of multimorbidity on mortality [19]. It was not possible to pool the existing data with regards to sex differences [19]. Multimorbidity measured as three or more long-term conditions increases specificity in older age [9,20]. Furthermore, requiring these multiple conditions to be present in separate body systems identifies multimorbidity that is likely to require care from several specialists, which has been termed complex multimorbidity [20]. Such complex multimorbidity presented a moderate relationship with mortality, adjusted for sociodemographic characteristics, in a study on individuals aged 60 to 69 years in Norway [21].

While various frailty measures identify distinct subgroups, all are associated with mortality [22]. Frailty consistently increased mortality risk when adjusted for multimorbidity and socioeconomic position in a population cohort aged 37 to 73 years [18]. Joint multimorbidity and frailty increased the mortality risk in older adults, while the separate measures did not [23]. Adjustment by socioeconomic position did not modify this relationship [23].

The relation of socioeconomic position with health outcomes vary by measure because each act through distinct mechanisms [24,25]. Modification of multimorbidity’s association with mortality has been explored by education [26,27], occupation [27], and deprivation indices [28,29]. Occupation is a comprehensive measure, reflecting income, material resources, and networks, as well as specific outcomes of biopsychosocial exposure on the job [25].

There are few studies examining the joint outcome of socioeconomic position and multimorbidity on mortality [26,27,28,29]. The studies vary in exploring one measure [27,28] or several measures [26,29] of multimorbidity and 1 measure [26,28,29] or several measures [27] of socioeconomic position throughout adulthood [26,28] or in restricted age groups [27,29] with a follow-up time ranging from 4 years [26] to 24 years [27]. Overall, they find multimorbidity is more common in those with social deprivations [26,28,29], while the association with subsequent mortality varies in presence [28,29] or absence [26,27] of sex differences and is reported as stable [29], reduced [26], and nonexistent [27] across socioeconomic strata.

In summary, multimorbidity and frailty share determinants, and like socioeconomic position, they are associated with mortality. There is a research gap in exploring the impact of multimorbidity, and possible pooled effect of multimorbidity and frailty [7] on mortality in various social strata and younger age groups [10].

Our aim was to explore how occupational position may modify the relationship between several measures of multimorbidity, including multimorbidity with frailty, and mortality in the general population. The study is conducted in a Nordic welfare state and results can be contrasted with similar studies conducted in a different welfare regime type model [30]. We report absolute and relative differences in all-cause mortality by occupational groups and sex and compare the prognostic value of different multimorbidity measures. We hypothesize that socioeconomic position will interact with all measures of multimorbidity and individuals in lower occupational groups will have worse prognoses.

## 2. Materials & Methods

### 2.1. Study Population and Sample

The Trøndelag Health Study (HUNT) is an ongoing population-based health study that invites all citizens of Trøndelag County, Norway, 20 years and older to participate. The current study used baseline data from the HUNT Study 2006–2008 (HUNT3), which invited a total of 93,860 individuals to participate. We report in accordance with Strengthening the Reporting of Observational Studies in Epidemiology (STROBE) guidelines for cohort studies [31]. Details on cohort profiles and data collection procedures have been published previously [32,33]. In short, participants were defined as responders to the main questionnaire, which was sent with the invitation by mail. Overall, 50,807 of 93,860 individuals (54.1%) participated in HUNT3 [32].

**Figure 1:**
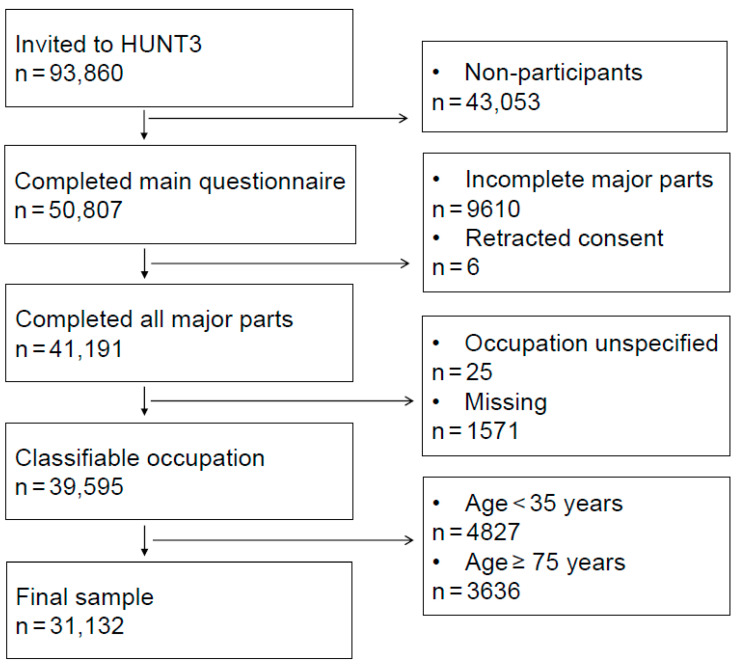
Flowchart for sample selection.

To be eligible for analysis in this cohort study, participants had to complete all major parts of HUNT3 (the main questionnaire, attend a screening station for interview, clinical measurements, and blood samples and return by mail a second questionnaire specific to age group and sex). Finally, classifiable occupational data, in addition to registry data (age, sex, and mortality status), were required. Figure 1 presents a flowchart for the sample selection. Individuals younger than 35 years were excluded upon expected low statistical power and to minimize the risk of misclassification by occupational group. Participants 75 years or older were omitted to minimize the effect of older adults in good health causing underestimation of occupational group differences in the association of multimorbidity with mortality.

### 2.2. Outcome Variable

#### All-Cause Mortality

The Norwegian National Population Registry administers all-cause mortality, from which the HUNT Databank regularly obtains registry status describing individuals in its cohort as being alive, having emigrated out of the country, or being dead. The registry data are linked on an individual level and there is no loss to follow-up. The last update from the National Population Registry and end of follow-up was 1 February 2019.

### 2.3. Independent Variables

#### Multimorbidity

We previously generated a set of 51 individual, chronic conditions from HUNT3 data [14,15] and further allocated those to body systems [14] by use of 14 chapters in the International Statistical Classification of Diseases 10th Revision (ICD-10) (supplementary data 1), hereafter called organ-grouped conditions.

From this set and categorization of chronic conditions, we created five multimorbidity measures of which two were continuous and three were categorical measures as follows:

(1) individual disease counts;
(2) organ-grouped disease counts;
(3) a threshold of three or more individual conditions;
(4) a threshold of three or more organ-grouped conditions called complex multimorbidity;
(5) co-occurrence of two or more individual conditions and frailty (measured as one of four dimensions (poor self-rated health, mental illness, physical impairment or social impairment)) called multimorbidity with frailty.

Both the multimorbidity and frailty measure included anxiety and depression. In total, 23 of 11,861 individuals met the criteria of two-condition multimorbidity plus one dimension of frailty, with the presence of anxiety and depression only.

Complex multimorbidity [20] and multimorbidity with frailty [34] are measures suggested to detect individuals in higher need of coordinated care which was previously operationalized in HUNT3 [14,15]. Organ-grouped disease counts were used in sensitivity analyses.

### 2.4. Sociodemographic Variables

Continuous age and categorical sex variables were provided by the HUNT Databank. Our proxy variable for socioeconomic position was occupation, derived from the interview question “What is/was the title of your main occupation?” Occupation is a comprehensive measure reflecting income, material resources, and networks, as well as specific biopsychosocial exposures on the job [25]. Occupations were categorized according to the simplified, 3-class version of the European Socio-economic Classification scheme (supplementary data 2) [35].

### 2.5. Statistical Analysis

To show the distribution of follow-up time and events, demographic factors, and baseline health characteristics across occupational groups, cross-tabulations were conducted. Numbers of affected individuals, percentages, and measures of central tendency and variability are presented.

Logistic, linear, and Cox regression models were used. First, logistic regression models were fitted separately for each sex and occupational group to study associations between the number of individual chronic conditions, entered as restricted cubic splines, and death in the following 10-year period. These models were adjusted for continuous age. Results from each model were subsequently combined in a joint graph showing mortality as estimated proportions (with 95% confidence intervals (CIs)) at age 60 years and presented for 0 to 12 individual chronic conditions. Joint graphs of the complete range of individual chronic conditions, as well as a sensitivity analysis in which the same method was used to study the association between the number of organ-grouped chronic conditions and 10-year risk of death and accompanying descriptive tables of frequencies and number of events by individual and organ-grouped conditions, are in supplementary data 3. The logistic regression model to study the associations of organ-grouped chronic conditions necessitated the inclusion of age squared. Second, to formally test if multimorbidity was modified by occupation, we specified linear regression models to investigate statistical interactions between continuous multimorbidity and occupation on an additive scale. We also fit models with statistical interactions between multimorbidity and sex. The threshold significance level was *p* < 0.05.

Finally, we modeled time to death using sex-stratified Cox proportional hazard models with a composite variable containing different combinations of multimorbidity (yes or no) and occupation (low, middle, or high). We used age measured in years as the time scale to either date of emigration, all-cause mortality, or end of follow-up (1 February 2019), whichever came first. We report hazard ratios with 95% CIs in forest plots. The number of deaths, total frequency, and proportions are listed by joint multimorbidity and occupational group measures, sex, and occupational groups in supplementary data 4.

All statistical analyses were done in Stata IC (StataCorp. 2019. Stata Statistical Software: Release 16. StataCorp LLC, College Station, TX, USA) and all visualizations were created with the user-written Stata coefplot command [36]. Data analysis took place from January to June 2020.

### 2.6. Patient and Public Involvement

Participants, patients, and stakeholders took part in the preparation of HUNT3. The data collection was completed from October 2006 to June 2008. At the time of designing this secondary analysis study, no patient groups represented the universal topic multimorbidity, and therefore no patient or public representatives were involved.

### 2.7. Ethics Statement

The Regional Committee for Medical and Health Research Ethics in Norway approved the current study (project no. 2014/2265).

## 3. Results

Included in the analyses were 31,132 of 50,807 HUNT3 participants (61.3%) with complete data on multimorbidity, work, and registry status (Figure 1), followed for a mean (standard deviation (SD)) of 11.1 (1.5) years (Table 1). Individuals were excluded because of withdrawn consent (*n* = 6), incomplete participation (*n* = 9610), unspecified occupation (*n* = 25), or missing occupational data (*n* = 1571), younger than 35 years (*n* = 4827) or 75 years or older (*n* = 3636). No individuals were excluded due to missing registry data. Sociodemographic characteristics for individuals who were ineligible or had missing data are presented in supplementary data 5.

**Figure 2:**
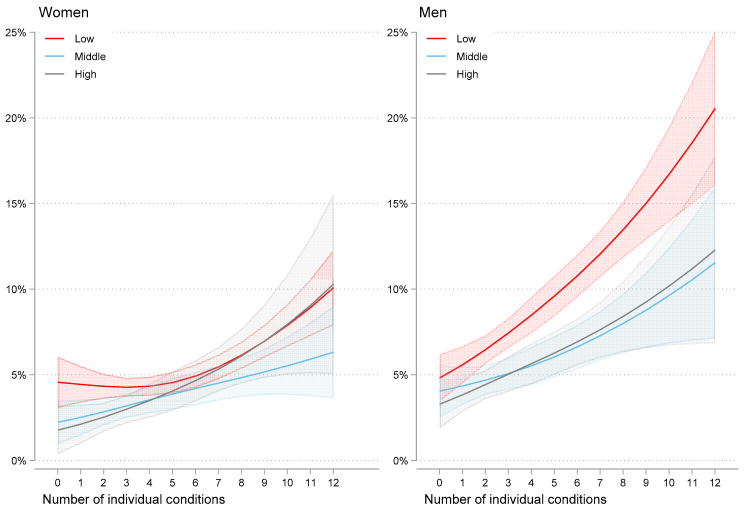
Estimated 10-year all-cause risk of death by number of individual long-term conditions and occupational group for women and men. Shading indicates 95% Cis. Low, middle, and high indicate occupational groups.

Nearly half the sample (15,261 of 31,132 (49.0%)) were designated as part of the low occupational groups, and a quarter (7501 of 31,132 (24.1%)) were in the high occupational group. The low occupational group had higher proportions of all measures of multimorbidity and reported a higher number of long-term conditions. A total of 2254 of 31,132 individuals (7.2%) died by the end of the study. By occupational group, this included 373 of 7501 (5.0%) in the high occupational group and 1310 of 15,261 (8.6%) in the low occupational group. Among the groups of multimorbidity, 1795 of 19,409 individuals (9.2%) with three or more individual conditions died, as did 1642 of 16,546 individuals (9.9%) with complex multimorbidity and 1312 of 11,861 individuals (11.1%) with multimorbidity and frailty (supplementary data 4). Risk of death according to occupation and number of individual chronic conditions are depicted for women and men in Figure 2.

Mortality increased by the number of individual chronic conditions in all occupational groups, but to varying degrees. For women, there was no clear tendency that occupation modified the association between the number of single conditions and mortality (additive statistical interaction *p* = 0.41), whereas for men, the low occupational group had a steeper increase than the middle and high occupational groups (additive statistical interaction *p* < 0.001). We also found evidence of a statistically significant interaction in which the number of conditions was more strongly associated with mortality for men compared with the same association in women (additive statistical interaction *p* = 0.03). A sensitivity analysis with multimorbidity measured as organ-grouped chronic conditions found the same statistical interactions and associations. In contrast to individual disease count, occupational differences in the risk of death in women were detectable for the full range of organ-grouped conditions (supplementary data 3, Figure S2).

**Figure 3:**
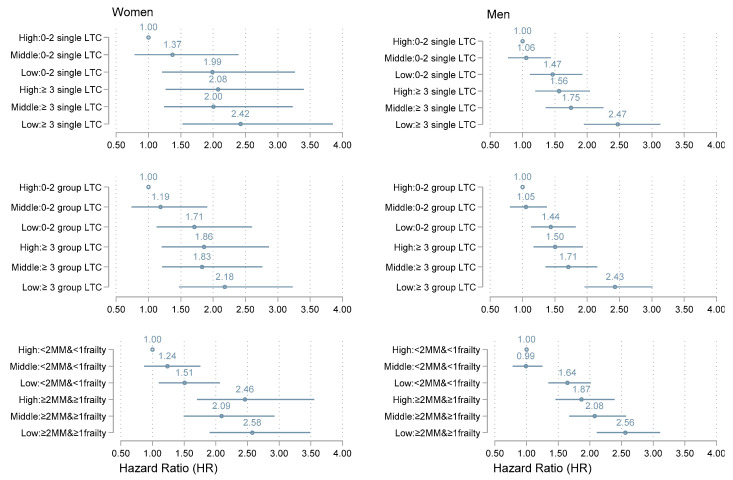
Hazard Ratios and 95% CIs for all-cause mortality between occupational groups with and without multimorbidity, separate for men and women. Abbreviations: LTC = long-term condition; MM, multimorbidity; 2MM & 1frailty = two individual long-term conditions plus one dimension of frailty (poor self-rated health, mental illness, physical impairment, or social impairment). Top panels, multimorbidity with a threshold of at least three individual long-term conditions; middle panels, multimorbidity as a threshold with at least three organ-grouped long-term conditions; bottom panel, multimorbidity with more than two individual long-term conditions and frailty.

Figure 3 shows hazard ratios (HRs) and 95% CI for mortality according to combinations of the categorical multimorbidity measures and occupation levels for women and men.

Compared with the reference category (individuals in the high occupation group whose health status was below the threshold of multimorbidity), the overall pattern suggests that the relative risk of death increased gradually with decreasing occupation levels and the presence of multimorbidity for both women and men. There was more than a two-fold risk of death in the low occupational group with all measures of multimorbidity compared with the reference category for both women and men.

## 4. Discussion

### 4.1. Summary of Findings

In this population cohort study on joint outcomes of socioeconomic position and multimorbidity on mortality, we found that all measures of multimorbidity and all-cause mortality were more common in lower occupational groups. Mortality increased with the number of chronic conditions, and occupational gradients were consistent. There was a tendency toward a stronger association between multimorbidity and mortality in men in lower occupational groups. Relative risk differences increased with lower occupational groups and the presence of multimorbidity.

### 4.2. Possible Mechanisms and Explanations

Socioeconomic differences in mortality result from unequal distribution of power, income, and resources [1]. Occupational position entails these general elements and particular outcomes of biopsychosocial exposures in the workplace [25], where negative factors tend to concentrate in lower occupational groups [1]. Multimorbidity may be associated with death through the lethality of each condition, interplay between conditions (including frailty), and conditions and treatments (such as polypharmacy and fragmented health care) [19]. The presence of frailty should initiate comprehensive, integrated care in patients with 2 or more individual conditions [34]. In this study, the relative risks were greatest with the presence of joint multimorbidity and frailty.

Because mortality increases with individual disease counts and any multimorbidity measure, a decrease in absolute and relative socioeconomic differences can be expected [37]. In women, absolute risk differences were diminished in the high occupational group, while the gradient between middle and low occupational groups persisted. This may imply heterogeneity in the multimorbidity measure, particularly in women. The occupational differences in mortality were greater by count of organ-grouped chronic conditions (supplementary data 3), and this may reflect that grouping by body system makes the measure more uniform and enables it to detect social gradients to a greater extent than simple disease counts. It seems that a continuous measure of multimorbidity better captures the impact of socioeconomic position on mortality.

### 4.3. Comparison with Existing Literature

In a review of 26 articles on the association between multimorbidity and mortality, all measures of multimorbidity increased mortality [19]. Three recent population cohorts, including 240,000 individuals [26] to 1.1 million individuals [28] and one worker cohort of 6425 people [27], have studied the joint outcome of socioeconomic position and multimorbidity on mortality. Two studies also explored the association of socioeconomic position with the transition from healthy to multimorbid [27,28] and frail [27]. Multimorbidity was measured as individual disease counts of 39 chronic conditions [26] or 43 chronic conditions [29] and as a threshold of 2 or more of 9 long-term conditions [27] and 30 long-term conditions [28]. Proxies for socioeconomic position were education [26,27], occupation [27], and area deprivation [28,29].

Similar to our findings, all [26,27,28,29] reported higher prevalence of multimorbidity in lower socioeconomic groups and more deaths with increasing multimorbidity and lower social position. With increasing disease count in a range of zero to more than four conditions, the impact of socioeconomic position on mortality were considered to decrease [26] or be stable [29]. The effect of increasing disease counts on mortality risk was larger among men [29], which corresponds to our findings. On the other hand, our study suggests increased mortality with consistent occupational gradients, with increasing disease counts for a greater range of individual conditions.

Socioeconomic position measured as occupation had the strongest association with onset of multimorbidity, physical frailty, and mortality [27]. In the presence of multimorbidity and frailty studied as separate measures, there were no social gradients in mortality [27]. In contrast, our study suggested intact occupational gradients in the presence of all categorical measures of multimorbidity.

Finally, in women with multimorbidity, life expectancy was equal across social strata, while men with multimorbidity had sustained social differences in survival [28]. Our findings also suggest that socioeconomic position can modify the association between multimorbidity and mortality in men but not in women.

We have not identified other studies of the outcome of socioeconomic position and joint multimorbidity and frailty on mortality. One study [23] reported the combination of multimorbidity and frailty to increase mortality risk, and adjustment by socioeconomic position resulted in no modification. In contrast, our stratified analysis on joint multimorbidity and frailty reveals occupational gradients in association with mortality.

Bias toward healthy older adults [26,28] and healthy workers [27] is likely in several of the studies, which will underestimate socioeconomic differences. Measuring multimorbidity as two individual chronic conditions has lower age specificity and detects smaller socioeconomic gradients than measures of organ-grouped conditions [9,20,38], which may impair the ability of such studies to detect socioeconomic differences in mortality. In sum, differences in setting and measures may explain variation in the impact of socioeconomic position and multimorbidity with mortality.

### 4.4. Strengths and Limitations

The HUNT Study is illustrative for Norway [33], and trends in the material match those of Western high-income countries [39,40,41]. To avoid a bias toward healthy older adults and misclassification of socioeconomic positions in younger age groups, we restricted the age range. Job opportunities and exposures in work may differ among birth cohorts; therefore, we adjusted by age and used age as the time scale in the analyses.

Occupation is an established comprehensive measure of socioeconomic position that may show stronger associations with health outcomes than unidimensional measures [27]. Occupational group classification enables international comparison [35].

There are no standard definitions or operationalizations of multimorbidity or frailty; however, self-report is a valid approach to measure multimorbidity in larger samples [9]. HUNT3 covers a broad range of conditions suitable to obtain proper estimates of multimorbidity [20]. We fitted HUNT3 data to a multidimensional frailty measure in agreement with a holistic, conceptual definition of frailty [42]. Registry data ensured no loss to follow-up and the ability to link outcomes on an individual level.

Our cohort study offers a unique opportunity to directly compare the outcome of occupational positioning and several multimorbidity measures on all-cause mortality. We compared mortality with reference groups that may have some morbidities, as recommended by a recent review on multimorbidity and mortality [19]. We reported absolute and relative mortality risk differences stratified by sex and socioeconomic position to clarify characteristics that may be useful to inform future interventions and are compliant with recommendations on reports of socioeconomic inequalities in health [43].

The participants in this study may to some extent have higher socioeconomic position and lower mortality compared with nonparticipants [44]. Further age restriction increased the proportion of individuals categorized in high occupational groups compared with the occupational groups of noneligible individuals. In sum, estimates of the association of multimorbidity and socioeconomic position with mortality will be conservative. Events are relatively few, and imprecision limits the interpretation of the results.

We only explored socioeconomic gradients by use of occupational positions. Various measures of socioeconomic position act through distinct mechanisms and associate differently with health outcomes [24,25]. Reverse causation, whereby prior health determines job opportunities, will increase detected differences. Our measure excludes those who had never worked, and older women are more likely to be missing because of unclassifiable occupations. This will probably underestimate social gradients [45].

To assess prospective health outcomes, there are recommendations to use weighted multimorbidity measures [46]. However, this was not possible with the data available. We lack information on chronicity for most conditions and may overestimate prevalence of multimorbidity. The multimorbidity and frailty measures are based on a count of conditions and dimensions and not types, which may vary among occupational groups. The heterogeneity may bias estimates in either direction. As for recognized frailty scales, our measure may differ in accuracy of detecting frailty across age groups [13,34]. This may underestimate outcome measures.

In this classic cohort study, we have measured multimorbidity at baseline. The duration of exposure prior to HUNT3 will vary by occupational group. A lack of updated measurements of health status may underestimate socioeconomic gradients [19].

### 4.5. Interpretation/Implication

Cautious of limitations and confounding, there was evidence of effect measure differences in mortality between occupational groups by the number of chronic conditions in this population cohort study, and these were stronger in men than women. Even complex measures of multimorbidity were prevalent. All multimorbidity measures were strongly associated with mortality with varying but consistent gradients between occupational groups and sex. It seems a continuous measure of organ-grouped multimorbidity would better capture the impact of socioeconomic positioning on mortality than categorical multimorbidity measures.

Norway is a high-income country with a well-developed welfare system. Primary and specialist health care are mostly public and financed through taxation with low costs for the individual, as are all levels of education. Job security and standards for health, safety, and environment in the workplace are high. The results can be transferable to similar welfare state models but can also be contrasted across different regime types. As others have noted, political systems and priorities shape population health and the magnitude of health inequities [30]. Observed marked differences in multimorbidity and mortality between occupational groups in our setting might suggest that labor protection legislation is important in all societies.

The results support that social differences in multimorbidity must be a priority in public health and should receive increased attention in health care. Improved management in the health care sector necessitates reforms to fit the complexity of multimorbidity, from research and education to clinicians and organization [34,47,48].

### 4.6. Future Research

The use of heterogenous multimorbidity measures in this study may obscure the relation to mortality and any socioeconomic modification thereof [10]. It may be advantageous to study clusters of multimorbidity to clarify causes and consequences [10]. Others have argued that clusters undermine that the norm is multiplicity, which is more than the sum of its morbidities [49,50]. Complex multimorbidity seems a relevant measure that captures this multiplicity while remaining sufficiently uniform to detect social differences in mortality.

On this background, we recommend exploration of complex multimorbidity as well as clusters of multimorbidity with repeated recordings and their association with a variety of socioeconomic position measures, health care utilization, and mortality, in an attempt to enhance future prevention and management of multimorbidity.

## 5. Conclusions

Multimorbidity is common and strongly associated with mortality with varying occupational gradients. Men in lower occupational groups seems to be a particularly vulnerable group. Prevention of multimorbidity is of public health importance in prolonging survival of all people. The health care sector, from workforce to organization, needs to enhance the generalist and person-centered focus sensitive to social context to better care for this large patient group. Continuous research on various measures of multimorbidity and associations to multiple sociodemographic variables, health care use, and mortality will be necessary to guide prevention and management.

## Figures and Tables

**Table 1:** Sociodemographic characteristics and health profile at baseline and follow-up time and events in occupational strata, the HUNT Study 2006–2008 (HUNT3).

Characteristics and Outcomes	Occupational Group							
	High		Middle		Low		Total	
Cohort, baseline, No. (%)	7501	(100)	8370	(100)	15,261	(100)	31,132	(100)
Women No. (%)	3702	(49.4)	4427	(52.9)	8821	(57.8)	16,950	(54)
Men No. (%)	3799	(50.6)	3943	(47.1)	6440	(42.2)	14,182	(46)
Age, years, mean (SD)	53	(10.2)	55	(10.7)	56	(10.5)	55	(10.5)
Health status, baseline								
Individual LTCs, median (IQR)	3	(1 to 5)	3	(2 to 5)	4	(2 to 6)	3	(2 to 5)
Organ-grouped LTCs, median (IQR)	2	(1 to 3)	3	(1 to 4)	3	(2 to 4)	3	(1 to 4)
≥3 individual LTCs, No. (%)	3919	(52.2)	5088	(60.8)	10,402	(68.2)	19,409	(62.3)
Complex multimorbidity, No. (%) ^a^	3191	(42.5)	4298	(51.4)	9057	(59.3)	16,546	(53.1)
Multimorbidity with frailty, No. (%) ^b,c^	2070	(27.6)	3081	(36.8)	6710	(44.0)	11,861	(38.1)
End of follow-up								
Follow-up time, years, mean (SD)	11.1	(1.3)	11.1	(1.5)	11.0	(1.6)	11.1	(1.5)
Person-years, thousands (%)	83.5	(24.3)	93.0	(27.0)	167.6	(48.7)	344.2	(100)
Deaths, No. (%)	373	(5.0)	571	(6.8)	1310	(8.6)	2254	(7.2)
Deaths in women, No. (%)	118	(31.6)	210	(36.8)	608	(46.4)	936	(41.5)
Deaths in men, No. (%)	255	(68.4)	361	(63.2)	702	(53.6)	1318	(58.5)
Age at death, years, mean (SD)	71.2	(8.7)	72.9	(8.2)	71.9	(8.6)	72.0	(8.5)

Abbreviations: No., number; SD, standard deviation; IQR, interquartile range; LTC, long-term condition. ^a^ Three or more organ-grouped LTCs. ^b^ Two or more individual LTCs and one dimension of frailty (poor health, mental illness, physical or social impairment). ^c^ In total, 15 people had data missing on frailty.

## Supplementary Materials

The following are available online at https://www.mdpi.com/2077-0383/9/9/2759/s1, Supplementary data 1: Construction of chronic, individual conditions and categorization; Table S1. 51 chronic conditions grouped by 14 ICD-10 chapters. Supplementary data 2: Details on operationalization of socioeconomic position; Table S2. The distribution of transformed occupational data and discrepancies between the Norwegian and International Standard Classifications of Occupations, and allocation in the European Socio-economic Classification scheme. Supplementary data 3: Estimated 10-year risk of death by all individual and organ-grouped chronic conditions, occupational groups and sex and descriptive data; Figure S1. Estimated 10-year all-cause risk of death by number of individual chronic conditions and cccupational group, women & men; Table S3. 10-year mortality by increasing individual chronic conditions, by sex and occupational strata; Figure S2. Estimated 10-year all-cause risk of death by number of organ-grouped chronic conditions and occupational group, women & men; Table S4. 10-year mortality by increasing organ-grouped chronic conditions, by sex and occupational strata. Supplementary data 4: Descriptive data supplementary to hazard ratios for mortality of combined occupational group and health status; Table S5. Number of deaths by occupational group and health status in women and men. Supplementary data 5: Baseline sociodemographic characteristics of eligible, non-eligible and missing.
