# Supplementary material for: Socioeconomic Position, Multimorbidity and Mortality in a Population Cohort: The HUNT Study
Source: J Clin Med. 2020 Aug 26;9(9):2759. doi: 10.3390/jcm9092759 (PMC7563449; doi:10.3390/jcm9092759)
Supplement: Supplementary file 1 [file jcm-09-02759-s001.pdf]

Supplementary data 1: Construction of chronic, individual conditions and categorization.

This appendix describes in detail the construction of 51 chronic, individual conditions from data in the HUNT3 Survey, by questionnaires and measurements. Table S1 presents the allocation of the conditions in organ-groups by use of 13 chapters in the International Statistical Classification of Diseases 10th Revision (ICD-10) and one chapter on symptoms and signs.

ORIGINAL QUESTIONNAIRE, ENGLISH VERSION

Main questionnaire

https://www.ntnu.edu/c/document_library/get_file?uuid=129b68c3-520c-457f-8b98-02c49219b2ee&groupId=140075

Sex- and age-specific questionnaire

https://www.ntnu.edu/c/document_library/get_file?uuid=35ae2816-4155-4b64-a259-770946fa46d4&groupId=140075

GENERAL COMMENTS

Chronicity

Chronicity was defined by either 1: duration (3 months or longer), 2: causing functional limitation (physical, mental, social) or 3: requiring health care management (pharmacological or not, primary or specialist care),[1] or 4: chronicity was assumed based on medical knowledge and clinical experience.

Missing

In variables with index questions and cluster text, missing was in general corrected for affirmed index question and regarded as "no" if replied to any alternative to any of the other questions in the block. Information on missing is also collected from the HUNT Databank.

References

References hold information on construction or accuracy of self-report of, or comparison of prevalence of the conditions to primary care and/or non-participant data. In general, self-report is considered to give reliable estimates of multimorbidity in studies of large samples.[2]

MAIN QUESTIONNAIRE

Hearing impairment[3]

Index question: "Do you suffer from longstanding (at least 1 year) illness or injury of a physical or psychological nature that impairs your functioning in your daily life?" Yes, no.

Options on follow-up question combined condition type (motor, vision, hearing, somatic, and psychiatric) and severity (slight, moderate, and severe).

Included with hearing impairment were those who reported chronic disease and moderate to severe hearing impairment.

"20 Diseases"

Cluster text: "Have you had or do you have any of the following;

Myocardial infarction;[4 5] angina pectoris;[5 6] heart failure;[4] other heart disease; stroke[4 5] or brain haemorrhage; kidney disease;[5] asthma;[5] chronic bronchitis, emphysema or chronic obstructive pulmonary disease; diabetes;[4 5] psoriasis;[7] eczema on hands; [8 9] cancer;[5 10] epilepsy;[11] rheumatoid arthritis;[5 12] ankylosing spondylitis;[5 12] sarcoidosis; osteoporosis;[5 13] fibromyalgia[5] and osteoarthritis[5]?"

Separate tick boxes for each diagnosis: Yes, no.

For each diagnosis, included were those who affirmed to have or have had the diagnosis.

Chronicity is assumed based on medical knowledge and clinical experience.

SEX- AND AGE-DIFFERENTIATED QUESTIONNAIRE

Headache[5]

Seven questions in one block. Question 1: "Have you had headaches in the last year?" Yes/no.

Migraine without aura[14]

Of those who affirmed headache last year, migraine without aura was constructed from three of seven questions:

1. "What is the average strength of your headaches?" 1=Mild, 2=Moderate, 3=Strong.

Recoded to dichotomous variable, where 1=Moderate/Strong.

2. "How long does the headache usually last?" 1=Less than 4 hours, 2=4 hours - 1 day, 3=1 - 3 days, 4= More than 3 days.

Recoded to dichotomous variable, where 1= Less than 4 hours – 3 days.

3. Cluster text: "Are the headaches usually characterized or accompanied by
   - Throbbing/thumping pain?" Yes, no.
   - Pain on one side of the head?" Yes, no.
   - Worsening with physical activity?" Yes, no.
   - Nausea and/or vomiting?" Yes, no.
   - Hypersensitivity to light and/or noise?" Yes, no.

Included with migraine: were those who affirmed to headache lasting 0 to 72 hours and at least two of four characteristics (pulsating quality, unilateral location, moderate/severe pain intensity, or aggravation by physical activity) and during headache having at least one of two accompanying symptoms (nausea and/or vomiting or increased sensitivity to light and/or noise).[14]

Chronicity is assumed based on medical knowledge and clinical experience.

Chronic headache14

Of those who affirmed headache last year, chronic headache was constructed from two of seven questions:

1. "If yes (headache in the last year): What type of headache? Migraine, other."

   The HUNT Databank created two variables with range 1: 1) migraine and 2) other headache.

2. "Average number of days a month with headaches:" 1=Less than 1 day, 2=1-6 days, 3=7-14 days, 4=More than 14 days.

Recoded to dichotomous variable, where 1= More than 14 days.

Included as case with chronic headache were those reporting "other" type of headache and an average frequency of more than 14 days per month.

Chronicity is assumed based on medical knowledge and clinical experience.Pain[5]

Index question: "In the last year, have you had pain or stiffness in muscles or joints that has lasted at least 3 consecutive months?" Yes, no.

The follow-up question "If yes: Where have you had this pain or stiffness?" was combined with a figure with arrows and tick boxes at nine locations (neck, upper back, lower back, shoulder, elbow, hand, hip, knee and ankle/foot).

Chronic widespread pain[15]

Dichotomous variables were made for each major body area: 1) Trunk (neck, upper and lower back), 2) Upper limb (shoulder, elbow, hand), and 3) Lower limb (hip, knee, foot/ankle), where 1=At least one painful location. A sum (row total) score variable was made for the major body areas and dichotomized, where 1=3, that is one pain in each major body area.

Of those who affirmed to pain or stiffness that has lasted more than three consecutive months, chronic widespread pain was defined as pain at more than three sites in all major body areas (trunk, upper and lower limbs) for more than three months in the last year.

Chronic, local pain

Of those who affirmed to pain or stiffness that has lasted more than three consecutive months, chronic, local pain was defined as pain in the neck or upper back or lower back or shoulder or elbow or hand or hip or knee or ankle/foot, excluding presence of chronic widespread pain, generating nine dichotomous variables.

Thyroidal disease[5]

Cluster text: "Has it ever been verified that you have/have had hypothyroidism or hyperthyroidism?" Separate tick boxes for each condition (yes, no), generating two dichotomous variables, 1=Yes.

For each diagnosis, included were those who affirmed to have or have had the diagnosis.

Chronicity is assumed based on medical knowledge and clinical experience.

Irritable bowel syndrome[16 17]

Index question: "Have you had stomach pain or discomfort in the last 12 months?" Answers: Yes, much; yes, a little; no. Irritable bowel syndrome was further constructed from four of six follow-up questions: "If yes:

"In the last 3 months, have you had this as often as 1 day a week for at least 3 weeks?" Yes, no.

"Is the pain/discomfort relieved by having a bowel movement?" Yes, no.

"Is the pain/discomfort related to more frequent or less frequent bowel movements than normal?" Yes,no.

"Is the pain/discomfort related to the stool being softer or harder than usual?" Yes, no.

Included with irritable bowel syndrome were those who affirmed little or much stomach pain or discomfort in the last year, who for as often as 1 day a week for at least 3 weeks in the last 3 months have had at least two of the following: pain/discomfort relieved by having a bowel movement, related to altered frequency of bowel movements, or related to altered stool appearance, resembling a modified version of the Rome criteria.[16 17]

Gastro-oesophageal reflux disease[5 18]

Cluster text: "To what degree have you had the following problems in the last 12 months?" Options combined type (nausea, heartburn/acid regurgitation, diarrhea, constipation, alternating constipation and diarrhea, and bloating) and frequency (never, a little, or much).

Generated one dichotomous variable, heartburn, where 1=Much.

Gastro-oesophageal reflux disease is defined as much heartburn/acid regurgitation in the last 12 months.[18]Anxiety[5 19]

Instrument variable: Hospital Anxiety and Depression Scale.[19] Every other statement of 14 statements covers symptoms on anxiety and depression and is scored 0-3. The HUNT Databank constructed a total score for anxiety (HADS-A), if all 7 anxiety items were answered.Anxiety was defined as HADS-A score >=8/21, indicating mild or possible anxiety.[20-22] Chronicity is assumed based on medical knowledge and clinical experience.Depression[5 19]

Instrument variable: Hospital Anxiety and Depression Scale.[19] Every other statement of 14 statements covers symptoms on anxiety and depression and is scored 0-3. The HUNT Databank constructed total score depression (HADS-D), if all 7 depression items were answered. Depression was defined as HADS-D score >=8/21, indicating mild or possible depression.[20-22] Chronicity is assumed based on medical knowledge and clinical experience.Chronic insomnia[5 23]

There were nine questions on sleeping pattern in one cluster, including three concerning insomnia. Initial text: "How often in the last 3 months have you

"Had difficulty falling asleep at night?" Never/seldom, sometimes, several times a week.

"Woken up repeatedly during the night?" Never/seldom, sometimes, several times a week.

"Woken too early and couldn't get back to sleep?" Never/seldom, sometimes, several times a week.

Chronic insomnia was defined as in the last 3 months, several times a week, having difficulty falling asleep at night and waking up repeatedly during the night, and waking up too early. A modified version of the diagnostic criteria for insomnia in the International Classification of Sleep Disorders.[23] Alcohol use disorder[24]

Instrument variable: Cut down/Annoyed/Guilty/Eye-opener, also known as the CAGE questionnaire.[24] The CAGE questionnaire is a 4-item scale with scores of 0-1. A summary variable was created and dichotomized in which a score of 1 indicates >=2 positive answers.

Alcohol use disorder was defined as CAGE score greater than 2.[25]

Chronicity is assumed based on medical knowledge and clinical experience.

Dental health problem

"How would you say your dental health is?" Very, bad, ok, good, very good.

Dental health problems were defined as self-reported bad or very bad dental health.[26]

Chronicity is assumed based on medical knowledge and clinical experience.

Menopausal hot flashes

Included: Women older than 30 years only.

Two questions were used to define menopausal illness:

"Do you have/have you had hot flashes due to menopause?" During the day, during the night, day and night, haven't had any.

"If you have had hot flashes, how would you describe them?" Very intense, moderately intense, hardly noticeable.

Included with menopausal hot flashes were those who reported hot flashes occurring daily and/or nightly and of at least moderate severity.

Chronicity is assumed based on medical knowledge[27] and clinical experience.Nocturia[28]

Excluded: Women and men, 20-29 years.

One question on nocturia, identical to that of the International Prostate Symptom Scale (IPSS), was asked to men and women older than 30 years.

"How many times do you get up during the night to urinate?" None, 1 time, 2 times, 3 times, 4 times, 5 times or more.

Nocturia was defined as two or more voids per night.[28]

Chronicity is assumed based on medical knowledge and clinical experience.Urine incontinence[5 29]

Excluded: men, 20-29 years.

Instrument variable: The Epidemiology of Incontinence in the County of Nord-Trøndelag (EPINCONT) questionnaire.[29]

Index question: Do you have involuntary loss of urine? Yes, no.

Urine incontinence was constructed from two of six follow up questions. "If yes":

"How often do you have involuntary loss of urine?" Less than once a month, once or more per month, once or more per week, every day and/or night

"How much urine do you leak each time?" Drops or little, small amount, large amounts.

Self-reported frequency and volume of leakage were multiplied to obtain the validated 4-level Sandvik Severity Index, categorizing incontinence as slight, moderate, severe, and very severe.[29]

Urine incontinence were included if severe to very severe.

Chronicity is assumed based on medical knowledge and clinical experience.Prostate symptoms[30 31]

Included: men older than 30 years only.

Instrument variable: The International Prostate Symptom Scale[30] was slightly modified in HUNT3,[31] becoming a 7-item scale with scores of 0-5 per question.

Included were prostate symptoms of at least moderate severity, i.e. summary score >= 8 points.[30]

Chronicity is assumed based on medical knowledge and clinical experience.

Eye diseases[32]

Excluded: men and women, 20-29 years.

Cluster text: "Do you have any of the following eye conditions?" Cataract, glaucoma, and macula degeneration. Separate tick boxes, yes, no.

For each diagnosis, included were those who affirmed to have or have had the diagnosis.

Chronicity is assumed based on medical knowledge and clinical experience.

MEASUREMENTS

Obesity[33 34]

HUNT Databank constructed the BMI variable, defined as (weight in kg)/(height in m2). Obesity was defined as either BMI>=35 or a BMI 25-34.9 and an increased waist circumference (>= 88 cm for females; >= 102 cm for males).[33 34]

Waist circumference had large inter-observer variation, https://hunt-db.medisin.ntnu.no/hunt-db/#/variable/28. The data was checked for outliers.

Chronicity is assumed based on medical knowledge and clinical experience.Hypertension[5]

Blood pressure in HUNT3 is measured three times at one consultation. The mean of measurement 2 and 3 is calculated by HUNT Databank. If measure 2 or 3 were missing, the other was used as estimate for the mean, https://hunt-db.medisin.ntnu.no/hunt-db/#/variable/144.

Hypertension was defined as measured mean systolic BP>= 180 mmHg or diastolic BP >= 110 mmHg or reporting use of antihypertensive medications, excluding self-reported cardiovascular disease, diabetes, or kidney disease, and excluding extreme measures.[26]

Chronicity is assumed based on medical knowledge and clinical experience.

Hypercholesterolemia[35]

Hypercholesterolemia was defined as total-cholesterol >= 8 mmol/L.[35]

Chronicity is assumed based on medical knowledge and clinical experience.

Table S1 51 chronic conditions grouped by 14 ICD-10 chapters

ICD-10 chapter

- Conditions

II Neoplasms

- Cancer

III Blood/blood-forming organs/immune mechanism

- Sarcoidosis

IV Endocrine/nutritional/metabolic

- Obesity
- Hypercholesterolemia
- Diabetes
- Hypothyroidism
- Hyperthyroidism

V Mental/behavioural

- Alcohol problem
- Depression
- Anxiety
- Insomnia

VI Nervous system

- Epilepsy
- Migraine
- Chronic headache, other

VII Eye/adnexa

- Cataract
- Macula degeneration
- Glaucoma

VIII Ear/mastoid

- Hearing impairment

IX Circulatory system

- Hypertension
- Angina pectoris
- Myocardial infarction
- Heart failure
- Other heart disease[1]

- Stroke or brain haemorrhage[1]

X Respiratory system

- Chronic bronchitis, emphysema or COPD[1,2]
- Asthma

XI Digestive system

- Dental health status
- Gastro-oesophageal reflux disease
- Irritable bowel syndrome

XII Skin/subcutaneous tissue

- Hand eczema
- Psoriasis

XIII Musculoskeletal/connective tissue

- Rheumatoid arthritis
- Osteoarthritis
- Ankylosing spondylitis
- Fibromyalgia
- Osteoporosis
- Local musculoskeletal pain/stiffness in:
    - Neck or upper back or lower back or
    - shoulder or elbow or hand or
    - hip or knee or foot/ankle

XIV Genitourinary system

- Kidney disease
- Urine incontinence
- Prostate symptoms
- Menopausal hot flashes

XVIII Symptoms/signs/abnormal clinical/laboratory findings

- Nocturia
- Chronic widespread pain

[1] An exception to single entity LTC.

[2] **COPD = Chronic Obstructive Pulmonary Disease.REFERENCES ON CONSTRUCTION OF CONDITIONS**

1. Goodman RA, Posner SF, Huang ES, et al. Defining and measuring chronic conditions: imperatives for research, policy, program, and practice. *Prev Chronic Dis* 2013;10:E66. doi: 10.5888/pcd10.120239 [published Online First: 2013/04/27]
2. Fortin M, Stewart M, Poitras ME, et al. A systematic review of prevalence studies on multimorbidity: toward a more uniform methodology. *Ann Fam Med* 2012;10(2):142-51. doi: 10.1370/afm.1337 [published Online First: 2012/03/14]
3. Valete-Rosalino CM, Rozenfeld S. Auditory screening in the elderly: comparison between self-report and audiometry. *Braz J Otorhinolaryngol* 2005;71(2):193-200. doi: /S0034-72992005000200013 [published Online First: 2006/02/01]

4. Okura Y, Urban LH, Mahoney DW, et al. Agreement between self-report questionnaires and medical record data was substantial for diabetes, hypertension, myocardial infarction and stroke but not for heart failure. *J Clin Epidemiol* 2004;57(10):1096-103.
5. Langhammer A, Krokstad S, Romundstad P, et al. The HUNT study: participation is associated with survival and depends on socioeconomic status, diseases and symptoms. *BMC Med Res Methodol* 2012;12:143. doi: 10.1186/1471-2288-12-143 [published Online First: 2012/09/18]
6. Merzenich H, Blettner M, Niehoff D, et al. Cardiac late events in German breast cancer patients: a validation study on the agreement between patient self-reports and information from physicians. *BMC Cardiovasc Disord* 2018;18(1):218. doi: 10.1186/s12872-018-0961-7 [published Online First: 2018/12/01]
7. Modalsli EH, Snekvik I, Asvold BO, et al. Validity of Self-Reported Psoriasis in a General Population: The HUNT Study, Norway. *J Invest Dermatol* 2016;136(1):323-5. doi: 10.1038/jid.2015.386 [published Online First: 2016/01/15]
8. Svensson Å, Lindberg M, Meding B, et al. Self-reported hand eczema: symptom-based reports do not increase the validity of diagnosis. *Br J Dermatol* 2002;147(2):281-84.
9. Meding B, Barregård L. Validity of self-reports of hand eczema. *Contact Dermatitis* 2001;45(2):99-103.
10. Navarro C, Chirlaque MD, Tormo MJ, et al. Validity of self reported diagnoses of cancer in a major Spanish prospective cohort study. *J Epidemiol Community Health* 2006;60(7):593-9. doi: 10.1136/jech.2005.039131 [published Online First: 2006/06/23]
11. Keezer MR, Bouma HK, Wolfson C. The diagnostic accuracy of screening questionnaires for the identification of adults with epilepsy: A systematic review. *Epilepsia* 2014;55(11):1772-80. doi: 10.1111/epi.12811
12. Videm V, Thomas R, Brown MA, et al. Self-reported Diagnosis of Rheumatoid Arthritis or Ankylosing Spondylitis Has Low Accuracy: Data from the Nord-Trondelag Health Study. *J Rheumatol* 2017;44(8):1134-41. doi: 10.3899/jrheum.161396 [published Online First: 2017/04/17]
13. Cunningham TD, DeShields SC. Factors associated with the accuracy of self-reported osteoporosis in the community. *Rheumatol Int* 2016;36(12):1633-40. doi: 10.1007/s00296-016-3573-5 [published Online First: 2016/10/08]
14. Hagen K, Zwart JA, Aamodt AH, et al. The validity of questionnaire-based diagnoses: the third Nord-Trondelag Health Study 2006-2008. *J Headache Pain* 2010;11(1):67-73. doi: 10.1007/s10194-009-0174-7 [published Online First: 2009/12/01]
15. Mundal I, Grawe RW, Bjorngaard JH, et al. Prevalence and long-term predictors of persistent chronic widespread pain in the general population in an 11-year prospective study: the HUNT study. *BMC Musculoskelet Disord* 2014;15:213. doi: 10.1186/1471-2474-15-213 [published Online First: 2014/06/22]
16. Hammer J, Talley NJ. Diagnostic criteria for the irritable bowel syndrome. *Am J Med* 1999;107(5A):5S-11S. doi: 10.1016/s0002-9343(99)00276-4 [published Online First: 1999/12/10]

17. Longstreth GF, Thompson WG, Chey WD, et al. Functional bowel disorders. *Gastroenterology* 2006;130(5):1480-91. doi: 10.1053/j.gastro.2005.11.061 [published Online First: 2006/05/09]
18. Ness-Jensen E, Lindam A, Lagergren J, et al. Changes in prevalence, incidence and spontaneous loss of gastro-oesophageal reflux symptoms: a prospective population-based cohort study, the HUNT study. *Gut* 2012;61(10):1390-7. doi: 10.1136/gutjnl-2011-300715 [published Online First: 2011/12/23]
19. Zigmond AS, Snaith RP. The hospital anxiety and depression scale. *Acta Psychiatr Scand* 1983;67(6):361-70. doi: 10.1111/j.1600-0447.1983.tb09716.x [published Online First: 1983/06/01]
20. Mykletun A, Stordal E, Dahl AA. Hospital Anxiety and Depression (HAD) scale: factor structure, item analyses and internal consistency in a large population. *Br J Psychiatry* 2001;179:540-4. doi: 10.1192/bjp.179.6.540 [published Online First: 2001/12/04]
21. Bjelland I, Dahl AA, Haug TT, et al. The validity of the Hospital Anxiety and Depression Scale. An updated literature review. *J Psychosom Res* 2002;52(2):69-77. doi: 10.1016/s0022-3999(01)00296-3 [published Online First: 2002/02/08]
22. Herrmann C. International experiences with the Hospital Anxiety and Depression Scale--a review of validation data and clinical results. *J Psychosom Res* 1997;42(1):17-41. doi: 10.1016/s0022-3999(96)00216-4 [published Online First: 1997/01/01]
23. Medicine AAoS. The international classification of sleep disorders: diagnostic and coding manual: American Acad. of Sleep Medicine 2005.
24. Ewing JA. Detecting alcoholism. The CAGE questionnaire. *JAMA* 1984;252(14):1905-7. doi: 10.1001/jama.252.14.1905 [published Online First: 1984/10/12]
25. Skogen JC, Overland S, Knudsen AK, et al. Concurrent validity of the CAGE questionnaire. The Nord-Trondelag Health Study. *Addict Behav* 2011;36(4):302-7. doi: 10.1016/j.addbeh.2010.11.010 [published Online First: 2010/12/21]
26. Tomasdottir MO, Getz L, Sigurdsson JA, et al. Co-and multimorbidity patterns in an unselected Norwegian population: Cross-sectional analysis based on the HUNT Study and theoretical reflections concerning basic medical models. *European Journal for Person Centered Healthcare* 2014;2(3):335-45. doi: https://doi.org/10.5750/ejpch.v2i3
27. Avis NE, Crawford SL, Greendale G, et al. Duration of menopausal vasomotor symptoms over the menopause transition. *JAMA Intern Med* 2015;175(4):531-9. doi: 10.1001/jamainternmed.2014.8063 [published Online First: 2015/02/17]
28. Tikkinen KA, Johnson TM, 2nd, Tammela TL, et al. Nocturia frequency, bother, and quality of life: how often is too often? A population-based study in Finland. *Eur Urol* 2010;57(3):488-96. doi: 10.1016/j.eururo.2009.03.080 [published Online First: 2009/04/14]
29. Sandvik H, Seim A, Vanvik A, et al. A severity index for epidemiological surveys of female urinary incontinence: comparison with 48-hour pad-weighing tests. *Neurourol Urodyn* 2000;19(2):137-45. doi: 10.1002/(sici)1520-6777(2000)19:2<137::aid-nau4>3.0.co;2-g [published Online First: 2000/02/19]

30. Barry MJ, Fowler FJ, Jr., O'Leary MP, et al. The American Urological Association symptom index for benign prostatic hyperplasia. The Measurement Committee of the American Urological Association. *J Urol* 1992;148(5):1549-57; discussion 64. doi: 10.1016/s0022-5347(17)36966-5 [published Online First: 1992/11/11]
31. HUNT Databank. International Prostate Symptom Scale in HUNT3 Questionnaire 2 [Webpage]. Levanger: HUNT Databank; 2019 [cited 2019 05.20.]. Available from: https://hunt-db.medisin.ntnu.no/hunt-db/#/instrumentpart/45_11 2019.
32. MacLennan PA, McGwin G, Jr., Searcey K, et al. Medical record validation of self-reported eye diseases and eye care utilization among older adults. *Curr Eye Res* 2013;38(1):1-8. doi: 10.3109/02713683.2012.733054 [published Online First: 2012/10/20]
33. Janssen I, Katzmarzyk PT, Ross R. Waist circumference and not body mass index explains obesity-related health risk. *Am J Clin Nutr* 2004;79(3):379-84. doi: 10.1093/ajcn/79.3.379 [published Online First: 2004/02/27]
34. Perreault L. Obesity in adults: Prevalence, screening, and evaluation. Waltham, MA: UpToDate 2018.
35. Helsedirektoratet. Nasjonal faglig retningslinje for individuell primærforebygging av hjerte-og karsykdommer, kortversjon, IS-1675. In: Helsedirektoratet, ed., 2009.

Supplementary data 2: Details on operationalization of socioeconomic position.

In the HUNT3 Survey interview, all participants were asked: "What is/was the title of your main occupation?" Free-text answers were manually classified according to the *Standard Classifications of Occupations* by Statistics Norway,[1] which is based on the European Union's version of the *International Standard Classification of Occupations-88*.[2]

The standard categorize occupations according to skill level and specialization, degree of independence, and manual labor but not social position.[1] Occupations are coded with up to four digits, with increasing detail. One digit indicates major groups; two digits, submajor groups; three digits, minor groups; and four digits, unit groups. The minor occupational group was the highest level of detail available in the HUNT3 Survey.

Occupational socioeconomic position was operationalized using the European Socio-economic Classification scheme.[3] The full version of the scheme requires employment status and size of organization in addition to occupation to assign a class position. We used the simplified class scheme, based on minor occupational group only[3], as the HUNT3 Survey did not have data corresponding to employment status and size of organization. It is shown that the agreement between three-digit full and simplified version of this scheme is 79.7% for the total workforce.[3]

The syntax is available from https://www.iser.essex.ac.uk/archives/esec/matrices-and-syntax. It was performed using SPSS 25.0 (SPSS Inc., Chicago, IL, USA).

Table S2 gives details of transformation of data, discrepancies between the Norwegian and European Union standard and the allocated position in the full classification scheme. 2179 individuals had alterations to their occupational data to fit the syntax, 5.7% (2179/38027) of the total sample.

In the HUNT3 Survey data, the minor occupational group was a string variable. To perform the syntax, it had to be altered to a numeric variable. The string "011" changed to numeric value "11," which was manually corrected in the syntax. Within the 3-digit variable, some participants were classified with 1 digit and 2 digits only. These were transformed to the corresponding 3-digit minor group, at the lowest level of detail, by manually adding suffix digits 0 or 00, which is allowed when information is insufficient (section 4.3)[1] and in line with operationalizing of European Socio-economic Classification (see footnote table 1).[3]

Norwegian minor groups, which were not found in the European Union standard, were altered to the level of detail in which corresponding groups could be identified. These were *Standard Classifications of Occupations* by Statistics Norway codes: 112 (corresponding to 2 digits), 25 (corresponding to 1 digit), 251-6 (corresponding to 1 digit), 349 (corresponding to 2 digits), 631 (corresponding to 1 digit), 641 (corresponding to 1 digit), 735 (corresponding to 2 digits), and 745 (corresponding to 2 digits).

In total, 9 classes were created. To increase power and simplify interpretation, the full scheme was collapsed into a 3-class version, with "high" combining class 1 and 2, "middle" combining 3 to 6, and "low" combining 7 to 9. [3] The high occupational class represents large employers, higher-grade and lower-grade professionals, administrative and managerial occupations, higher-grade technician occupations, and supervisory occupations. The middle occupational class consist of small employers, self-employed individuals, lower supervisory occupations, and lower technician occupations. The low occupational class contain lower services, sales and clerical occupations, lower technical occupations, and routine occupations.

Table S2. The distribution of transformed occupational data and discrepancies between the Norwegian and International Standard Classifications of Occupations, and allocation in the European Socio-economic Classification scheme.

**Standard Classifications of Occupations**		**European Socio-economic**		
**Norwegian**	**International**	**Classification scheme**	***n***	**%**

1	100	1	262	(0.69)
011 (=num 11)	011=11	3	134	(0.35)
**112***	11=110	1	31	(0.08)
12	120	1	73	(0.19)
13	130	4	20	(0.05)
2	200	1	10	(0.03)
21	210	1	10	(0.03)
22	220	1	1	(0.00)
23	230	2	27	(0.07)
24	240	1	9	(0.02)
25	2=200	1	4	(0.01)
**251***	2=200	1	296	(0.78)
**252***	2=200	1	48	(0.13)
**253***	2=200	1	20	(0.05)
**254***	2=200	1	138	(0.36)
**255***	2=200	1	64	(0.17)
**256***	2=200	1	46	(0.12)
3	300	3	39	(0.10)
31	310	2	37	(0.10)
33	330	3	241	(0.63)
34	340	3	45	(0.12)
**349***	34=340	3	160	(0.42)
4	400	3	1	(0.00)
41	410	3	1	(0.00)
42	420	3	1	(0.00)
5	500	7	1	(0.00)
51	510	7	8	(0.02)
61	610	5	4	(0.01)
**631***	→6=600	5	93	(0.24)
**641***	→6=600	5	99	(0.26)
7	700	8	20	(0.05)
71	710	8	1	(0.00)
72	720	8	6	(0.02)
73	730	6	1	(0.00)
**735***	→73=730	6	38	(0.10)
74	740	8	1	(0.00)
**745***	→74=740	8	46	(0.12)
8	800	9	62	(0.16)
81	810	9	38	(0.10)
82	820	9	35	(0.09)
83	830	9	6	(0.02)
9	900	9	1	(0.00)
93	930	9	1	(0.00)
**Sum**			2179	(5.73)

Bold* = Divergence of Standard Classifications of Occupations by Statistics Norway from the European Union's version of The International Standard Classification of Occupations-88.

1. Statistics Norway. *Standard Classification of Occupations*. Oslo/Kongsvinger: Statistics Norway, 1998.
2. International Labour Organization (ILO). The International Standard Classification of Occupations, ISCO-88, https://www.ilo.org/public/english/bureau/stat/isco/isco88/index.htm (1988, accessed 24.05. 2019).
3. Rose D and Harrison E. The european socio-economic classification: A new social class schema for comparative European research. *Eur Soc* 2007; 9: 459-490. DOI: 10.1080/14616690701336518.

Supplementary data 3: Estimated 10-year risk of death by all individual and organ-grouped chronic conditions, occupational groups and sex and descriptive data.

Joint effect of occupational group and multimorbidity on 10-year mortality were presented in the article visualized by splines regression of individual chronic conditions for each occupational group and by sex at age 60 years. This appendix presents supplemental visual presentation of the full range 0 to 21 individual chronic conditions (figure S1). In a sensitivity analysis, the full range 0 to 11 organ-grouped chronic conditions was graphed by use of the same method (figure S2).

The accompanying total frequencies and number of events are presented in Table S3 for individual chronic conditions and Table S4 for organ-grouped chronic conditions, by occupational group and sex.

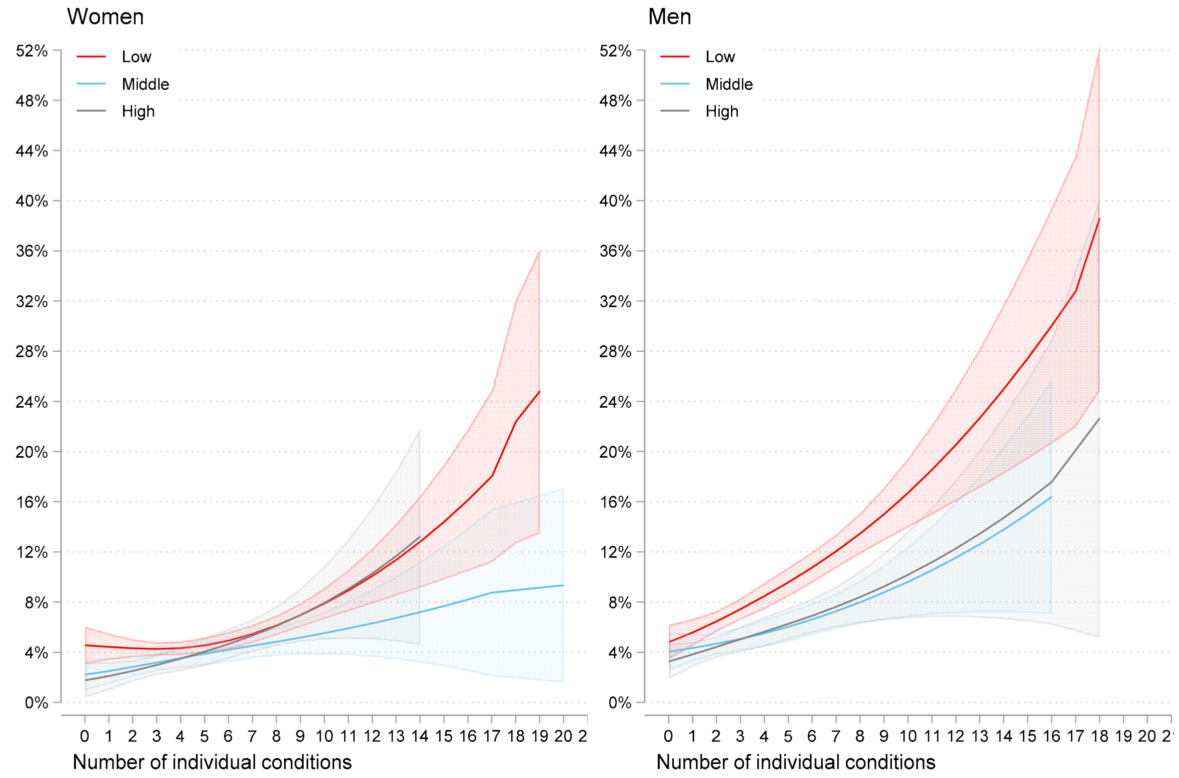

Figure S1. Estimated 10-year all-cause risk of death by number of individual chronic conditions and occupational group, women & men.

Shading indicates 95% CIs; low, middle, and high indicate occupational groups.

Table S3. 10-year mortality by increasing individual chronic conditions, by sex and occupational strata.

	**Women**						Men					
**Occ.group/**	**High**		**Middle**		Low		High		Middle		Low	
Individual CC	Deaths	Freq.	Deaths	Freq.	Deaths	Freq.	Deaths	Freq.	Deaths	Freq.	Deaths	Freq.
0	2	367	5	309	9	367	13	563	16	455	16	627

1	5	587	8	582	23	770	19	782	13	617	41	943
2	7	619	12	644	50	1138	28	664	40	675	60	1014
3	17	545	15	642	40	1224	31	556	45	587	67	928
4	6	453	23	553	39	1170	30	414	39	530	74	808
5	16	384	22	455	63	1098	23	295	25	345	73	672
6	13	273	16	420	66	909	19	186	30	271	61	513
7	6	175	20	296	45	664	10	124	20	166	48	333
8	4	114	12	210	47	572	10	93	15	108	47	222
9	6	77	11	123	25	359	5	48	12	72	26	141
10	4	47	7	81	19	218	7	29	11	49	17	90
11	7	32	6	48	13	138	2	16	3	22	16	59
12	3	17	3	38	12	91	2	15	3	21	10	42
13	1	6	3	10	9	41	2	5	3	11	8	22
14	2	6	0	5	6	28	1	3	2	8	6	11
15			1	5	7	19	2	3	2	2	4	9
16			1	3	2	10	0	2	1	4	1	3
17			0	2	0	2					0	1
18			0	1								
19					1	2	0	1			2	2
20					1	1						
Total	99	3702	165	4427	477	8821	204	3799	280	3943	577	6440

Abbreviations: CC=chronic conditions.

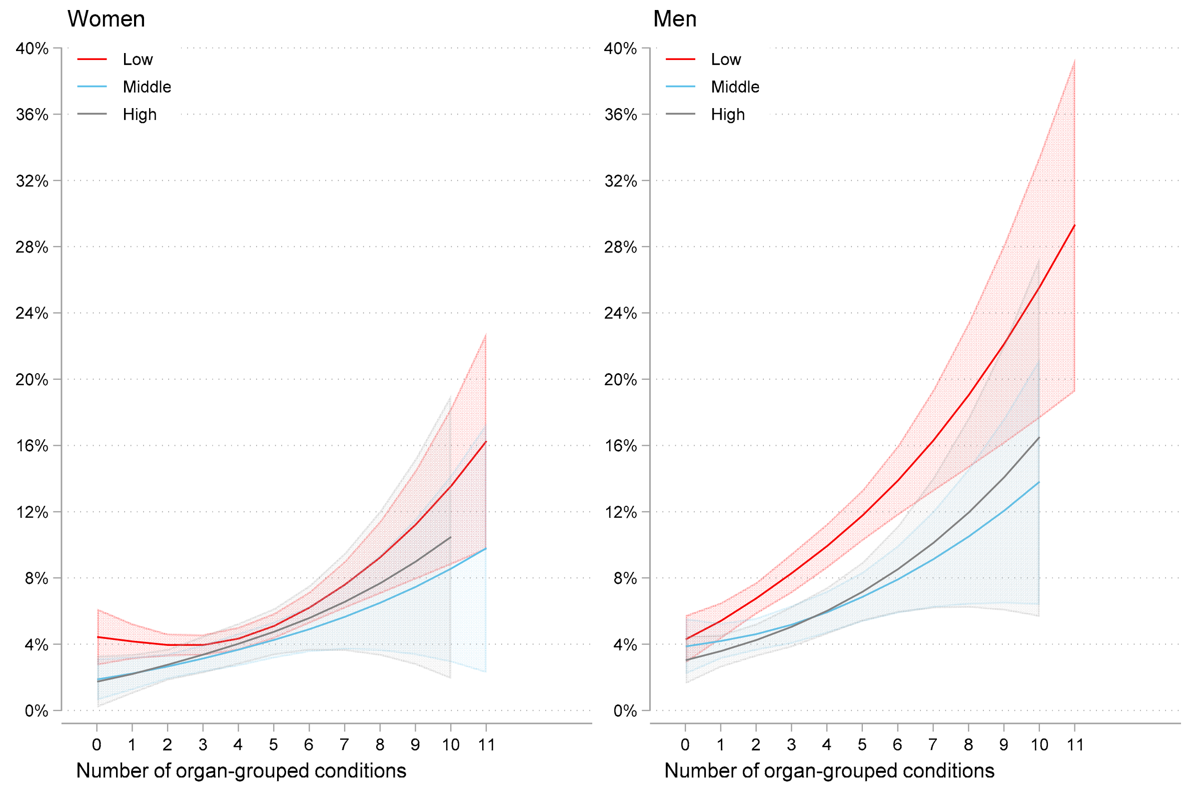

Figure S2. Estimated 10-year all-cause risk of death by number of organ-grouped chronic conditions and occupational group, women & men.

Shading indicates 95% CIs; low, middle, and high indicate occupational groups.

Table S4. 10-year mortality by increasing organ-grouped chronic conditions, by sex and occupational strata.

	Women						Men					
**Occupational group/**	High		Middle		Low		High		Middle		Low	
Organ-grouped CC	Deaths	Freq.	Deaths	Freq.	Deaths	Freq.	Deaths	Freq.	Deaths	Freq.	Deaths	Freq.
0	2	367	5	309	9	367	13	563	16	455	16	627
1	10	745	8	719	31	985	23	941	24	790	55	1205
2	9	823	19	893	63	1622	41	871	51	906	84	1398
3	19	738	30	881	68	1847	45	652	65	724	105	1210
4	18	452	31	668	90	1499	35	389	47	530	109	916
5	20	290	26	488	79	1152	22	215	31	270	80	530
6	9	177	25	266	68	747	8	97	19	137	63	318
7	7	69	12	120	35	392	10	48	11	78	35	141
8	5	30	6	61	20	141	2	12	10	39	19	66
9	0	10	1	15	10	49	5	9	4	10	6	19
10	0	1	2	6	4	18	0	2	2	4	4	7
11			0	1	0	2					1	3
Total	99	3702	165	4427	477	8821	204	3799	280	3943	577	6440

Abbreviations: CC=chronic conditions.

Supplementary data 4: Descriptive data supplementary to hazard ratios for mortality of combined occupational group and health status.

Table S5 lists frequency and proportions of deaths by those fulfilling and not fulfilling the criteria(s) of the categorical multimorbidity measures, occupational strata and sex. The multimorbidity measures were; more than three single long-term conditions (LTC), more than three organ-grouped LTC and two-condition multimorbidity plus one dimension of frailty.

Table S5. Number of deaths by occupational group and health status in women and men.

**Multimorbidity**	Occupational group	Women Freq.	Deaths	(%)	Men Freq.	Deaths	(%)
≥3 single LTC							
No	High	1573	19	(1.2)	2009	79	(3.9)
	Middle	1535	36	(2.3)	1747	84	(4.8)
	Low	2275	93	(4.1)	2584	148	(5.7)
Yes	High	2129	99	(4.7)	1790	176	(9.8)
	Middle	2892	174	(6.0)	2196	277	(12.6)
	Low	6546	515	(7.9)	3856	554	(14.4)
≥3 group LTC							
No	High	1935	27	(1.4)	2375	102	(4.3)
	Middle	1921	46	(2.4)	2151	114	(5.3)
	Low	2974	123	(4.1)	3230	200	(6.2)
Yes	High	1767	91	(5.1)	1424	153	(10.7)
	Middle	2506	164	(6.5)	1792	247	(13.8)
	Low	5847	485	(8.3)	3210	502	(15.6)
≥2MM&≥1frailty							
No	High	2623	48	(1.8)	2805	137	(4.9)
	Middle	2758	89	(3.2)	2530	141	(5.6)
	Low	4704	222	(4.7)	3836	300	(7.8)
Yes	High	1078	70	(6.5)	992	117	(11.8)
	Middle	1668	120	(7.2)	1413	220	(15.6)
	Low	4108	384	(9.3)	2602	401	(15.4)

Supplementary data 5:

Table S6. Baseline sociodemographic characteristics of eligible, non-eligible and missing.

	Sample		Non-eligible		Missing		Total	
	Freq.	(%)	Freq.	(%)	Freq.	(%)	Freq.	(%)
Total	31132	(100)	18097	(100)	1577	(100)	50806	(100)
Occupational group								
High	7501	(24.1)	3395	(18.8)	0	(0)	10896	(21.5)
Middle	8370	(26.9)	4153	(23.0)	0	(0)	12523	(24.7)
Low	15261	(49.0)	9360	(51.7)	1	(0)	24622	(48.5)
Missing	0	(0.0)	1189	(6.6)	1576	(100)	2765	(5.4)
Sex								
Women	16950	(54.5)	9524	(52.6)	1281	(81.2)	27755	(54.6)
Men	14182	(45.6)	8573	(47.4)	294	(18.6)	23049	(45.4)
	Median	(IQR)	Median	(IQR)	Median	(IQR)	Median	(IQR)
Age	55.4	(35.0 to 74.9)	43.5	(19.1 to 96.9)	71.4	(25.1 to 94.4)	53.6	(41.3 to 96.9)
